# Controlled clinical trial comparing the effectiveness of a mindfulness and self-compassion 4-session programme versus an 8-session programme to reduce work stress and burnout in family and community medicine physicians and nurses: MINDUUDD study protocol

**DOI:** 10.1186/s12875-019-0913-z

**Published:** 2019-02-06

**Authors:** Luis-Angel Pérula-de Torres, Juan Carlos Verdes-Montenegro Atalaya, Javier García-Campayo, Ana Roldán-Villalobos, Rosa Magallón-Botaya, Cruz Bartolomé-Moreno, Herminia Moreno-Martos, Elena Melús-Palazón, Norberto Liétor-Villajos, Francisco Javier Valverde-Bolívar, Nur Hachem-Salas, Luis-Alberto Rodríguez, Mayte Navarro-Gil, Ronald Epstein, Antonio Cabezón-Crespo, Carmen Morillo-Velarde Moreno, the collaborative group of the MINDUUDD study

**Affiliations:** 1 Clinical and Epidemiological Research Group in Primary Care (GICEAP), IMIBIC/Reina Sofía University Hospital/University of Córdoba, Primary Care Prevention and Health Promotion Research Network (RedIAPP), Family and Community Medicine Teaching Unit of Córdoba, Córdoba, Spain; 2 Comuneros Health Center, Research Network on Communication and Health (RICyS) of the Communication and Health program (semFYC), Burgos, Spain; 3 Primary Care Prevention and Health Promotion Research Network (RedIAPP), Centre for Biomedical Research Network on Mental Health, Zaragoza, Spain; 4 Miguel Servet Hospital, University of Zaragoza, Zaragoza, Spain; 5 Clinical and Epidemiological Research Group in Primary Care (GICEAP), IMIBIC/Reina Sofía University Hospital/University of Córdoba. Family and Community Medicine Teaching Unit of Córdoba, Córdoba, Spain; 6 Arrabal Health Centre. Primary Care Prevention and Health Promotion Research Network (RedIAPP), University of Zaragoza, Health Research Institute of Aragón, Zaragoza, Spain; 7 Family and Community Care Teaching Unit of Zaragoza Sector I, Zaragoza, Spain; 8 UGC Almería Periphery. Retamar Health Center, Research Network on Communication and Health (RICyS) of the Program Communication and Health (semFYC), Almería, Spain; 9 Belén Health Centre, Jaén, Spain; 10 Family and Community Medicine Teaching Unit of Jaén, Jaén, Spain; 11 Mediterranean Health Centre, Torrecárdenas, Almería, Spain; 12 Familiar and Community Attention Multiprofessional Teaching Unit of León II, Ponferrada, León, Spain; 13 Department of Psychology and Sociology, University of Zaragoza, Zaragoza, Spain; 14 University of Rochester Medical Center, New York, USA; 15 Family and Community Medicine Teaching Unit of Burgos, Burgos, Spain; 16 Family and Community Medicine Teaching Unit of Córdoba. Lucena II Health Centre, Córdoba, Spain

## Abstract

**Background:**

Health personnel are susceptible to high levels of work stress and burnout due to the psychological and emotional demands of their work, as well as to other aspects related to the organisation of that work. This paper describes the rationale and design of the MINDUUDD study, the aim of which is to evaluate the effectiveness of a mindfulness and self-compassion 4-session programme versus the standard 8-session programme to reduce work stress and burnout in Family and Community Medicine and Nursing tutors and residents.

**Methods:**

The MINDUDD study is a multicentre cluster randomised controlled trial with three parallel arms. Six Teaching Units will be randomised to one of the three study groups: 1) Experimental Group-8 (EG8); 2) Experimental Group-4 (EG4) Control group (CG). At least 132 subjects will participate (66 tutors/66 residents), 44 in the EG8, 44 in the EG4, and 44 in the CG. Interventions will be based on the Mindfulness-Based Stress Reduction (MBSR) program, including some self-compassion practices of the Mindful Self-Compassion (MSC) programme. The EG8 intervention will be implemented during 8 weekly face-to-face sessions of 2.5 h each, while the EG4 intervention will consist of 4 sessions of 2.5 h each. The participants will have to practice at home for 30 min/day in the EG8 and 15 min/day in the EG4. The Five Facet Mindfulness Questionnaire (FFMQ), Self-Compassion Scale (SCS), Perceived Stress Questionnaire (PSQ), Maslach Burnout Inventory (MBI), Jefferson Scale of Physician Empathy (JSPE), and Goldberg Anxiety-Depression Scale (GADS) will be administered. Measurements will be taken at baseline, at the end of the programs, and at three months after completion. The effect of the interventions will be evaluated by bivariate and multivariate analyses (Multiple Linear Regression).

**Discussion:**

If the abbreviated mindfulness programme is at least as effective as the standard program, its incorporation into the curriculum and training plans will be easier and more appropriate. It will also be more easily applied and accepted by primary care professionals because of the reduced resources and means required for its implementation, and it may also extend beyond care settings to academic and teaching environments as well.

**Trial registration:**

The study has been registered at ClinicalTrials.gov (NCT03629457; date of registration: 13.08.2018).

**Electronic supplementary material:**

The online version of this article (10.1186/s12875-019-0913-z) contains supplementary material, which is available to authorized users.

## Background

Work stress and burnout are two common problems among the health personnel of the Spanish National Health System *(Sistema Nacional de Salud-SNS)* due to the high health care burden on this system, among other reasons, with users increasingly demanding solutions to their health problems and needs, insufficient time for training and recycling, and perceived lack of support from managers [1–3]. Many studies show the need to address the physical and psychological consequences of work stress and burnout on health professionals not only by implementing measures regarding work organisation and working conditions but also by providing professionals with tools promoting self-care to help them cope with reality through emotional self-regulation [4, 5].

The Spanish model for training physicians and nurses in different health specialities—known as the *Internal Medical Resident Specialisation Program (MIR)* [6]—is of high quality and is highly regarded in Europe and Latin America. The speciality of Family and Community Medicine (via MIR) is 4 years and that of Family and Community Nursing (via EIR) is 2 years. Both specialities have been developed under the organisation and structure of the Teaching Units (TU) of Family and Community Medicine and Nursing within the SNS. Although residents are expected to assume responsibilities progressively, work stress is inevitable, especially during emergency shifts. An important figure in resident training is the personal tutor. This is a Primary Care (AP) professional with a minimum of five years of experience in patient care who voluntarily and selflessly tutors residents in training. This overload involving patient care and resident supervision generates more work stress and burnout.

Mindfulness is considered a third-generation therapy [7], having attained a strong reputation in the last several decades in Western countries, especially following the development of the MBSR technique by Jon Kabat-Zinn at the University of Massachusetts (USA) [8–10].

Since 2016, in countries such as the USA, at least 30% of medical schools have already included mindfulness in their curriculum plans [7], but in Spain, programmes to reduce work stress and burnout—both in undergraduate and postgraduate studies and in health care settings—by encouraging this practice are still rare. A study conducted by our group found that few AP professionals know of and practice mindfulness [11], even though authors are increasingly recommending training interventions in this technique [12]. Several studies show that by enhancing self-awareness, the professional improves self-care, which improves psychological well-being. Mindfulness-based interventions show good results in improving coping with stress and burnout and in increasing empathy in professionals [13–15], including those dedicated to health care [16].

The term compassion refers to “a feeling of affection or closeness towards other human beings who are suffering” [17–19], an aspect of vital importance in patient care. A key element of compassion for health professionals is self-compassion. If professionals are not accepted and respected, they will hardly display those emotions towards the people to whom they lend their help through empathy [20]. Self-compassion is often linked to mindfulness and is usually incorporated into training programmes [21] because it has been shown to facilitate doctor-patient communication and to improve clinical and psychological patient parameters [22, 23].

Few previous studies allow us to assess the effect of mindfulness programmes and self-compassion therapy in reducing stress and burnout in AP health personnel. The existing studies [16, 24, 25], together with a systematic review of 8 studies including health professionals and teachers, show the favourable effect of these programmes and strong evidence of the use of mindfulness practice to reduce burnout.

The most well-known mindfulness training program, the MBSR [8–10], consists of 8 weekly sessions lasting 120–150 min, which requires a high level of student commitment to adhere to the sessions and to devote 45 min of daily practice at home. Several attempts have been made to shorten the programs, mostly proposing to reduce interventions to four weeks to make them more viable and accessible while trying to maintain their therapeutic effects and effectiveness and adapting them to the specific target populations and contexts [26–29]. We have found no systematic reviews on the efficacy of these shorter interventions compared to the standard MBSR-based programmes when applied to health professionals—both to attending physicians and to those in postgraduate training. A recently published study [30] concluded that both programmes work in the same way, although the recruited population only included university students. Therefore, it is necessary to undertake studies providing us with greater and more robust evidence on the effectiveness of the abbreviated mindfulness and self-compassion programmes in the group of AP professionals, which will allow us to recommend the inclusion of such programmes in the postgraduate curriculum and in continuing education programmes.

## Objective

The objective of the MINDUUDD study is to evaluate the effect of a mindfulness and self-compassion training programme on the levels of work stress and burnout in residents and tutors of Family and Community Medicine and Nursing. This study is based on the hypothesis that an abbreviated 4-session programme of mindfulness and self-compassion is at least as effective as the standard 8-session programme to increase mindfulness and reduce the levels of work stress and burnout of AP residents and tutors.

## Methods/design

### Study design (Fig. 1)

The MINDUUDD study comprises an open-label, pragmatic, non-inferiority, multicentre, cluster randomised controlled trial with three parallel arms. The participating UUDDs will be randomised to one of the three study groups. Interventions will last for 8 weeks for one group (EG8) and 4 weeks (EG4) for the other. In both groups, there will be a follow-up at three months to check the maintenance of the programs’ effect. The control group (CG) will be reassessed 8 weeks after the start of the fieldwork.

**Fig. 1:**
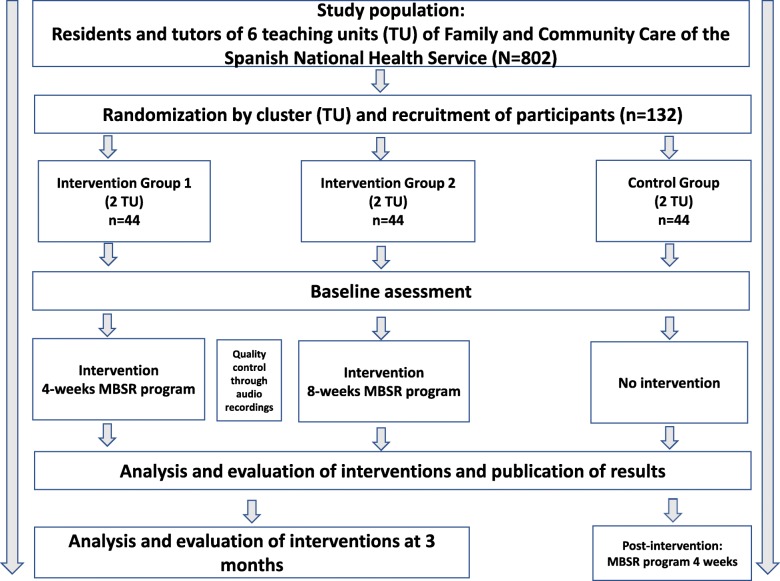
Cluster-randomised trial intervention scheme

### Study setting

The study population will be AP professionals who work in the SNS, assigned to 6 TU (total *N* = 802, Córdoba = 256, Almería = 147, Jaen = 185, Burgos = 64, Ponferrada = 63, and Zaragoza Sector I = 87), whether AP tutors (*N* = 297) or residents (*N* = 595) of Family and Community Medicine and Nursing.

### Eligibility criteria

-Inclusion criteria: AP professionals who are active and give their informed consent to participate in the study.

-Exclusion criteria: Having previously attended a mindfulness training course or workshop of at least 4 weeks, being an active mindfulness practitioner, being on prolonged sick leave during fieldwork, or having mental disorders discouraging the intervention.

### Intervention scheme

The interventions will be based on the MBSR programme [8–10] designed by Jon Kabat-Zinn and used at the Medical Center of the University of Massachusetts. Certain self-compassion practices of the Mindful Self-Compassion (MSC) programme [17–19] (Table 1) will be incorporated. The programmes to be tested will be adapted to the participating health care group and will differ by their duration and time dedicated to the tasks [30]:

1. Standard program: The format will be 8 weekly sessions of 2.5 h each. Participants must practice at home for 30 min a day.
2. Abbreviated program: The format will be 4 weekly sessions of 2.5 h each. Participants must practice at home for 15 min a day.

**Table 1:** Mindfulness and self-compassion training programs

	8-WEEK PROGRAM	4-WEEK PROGRAM
PRESENTATION BASELINE MEASUREMENT	General explanation, objectives, schedule. Pre-intervention measurements: general data, FFMQ, SCS, PSQ, MBI-GS, JSPE, GADS, AES questionnaires.	
1st SESSION	Raisin exercise. What is mindfulness? Practice: body scan. Informal practice: brushing.teeth Task: 30′ body scan.	Raisin exercise. What is mindfulness? Managing thoughts and emotions during practice. Practice: body scan. Informal practice: brushing.teeth Task: 30′ body scan.
2nd SESSION	Concepts: stress and burnout Practice: mindful breathing (sitting). 3-min mindfulness practice. Informal practice: mindful shower, 3′. Task: 30′ body scan + 10′ sitting.	Concepts: stress and burnout. Primary and secondary suffering. Practice: mindful breathing (sitting). 3-min mindfulness practice. Informal practice: mindful shower, 3′. Task: 30′ body scan + 10′ sitting.
3rd SESSION	Posture. Being versus doing. Managing thoughts and emotions during practice. Practice: yoga (standing meditation). Informal practice: washing the dishes, 3′. Task: alternating body scan or yoga + 15′ sitting.	Unconditional love (metta). Compassion. Practice: conscious movements. Metta for oneself and for others. Informal practice: washing the dishes, 3′, thank you letter. Task: alternating body scan or yoga + 15′ sitting.
4th SESSION	Attention. Primary and secondary suffering. Practice: yoga (sitting meditation). Practice: making the bed, 3′. Task: alternating body scan or yoga + 15′ sitting.	Positive psychology. Values. Practice: walking meditation. The funeral. Informal practice: the first bite, 3′, 3 positive things. Course feedback. Incorporating mindfulness into daily life. Course evaluation and satisfaction questionnaire. Repeating the questionnaires.
5th SESSION	Time management. Problem resolution. Practice: walking meditation. Practice: the first bite. Task: yoga alternating with 30′ sitting. Start walking meditation.	
6th SESSION	Unconditional love (metta). Compassion. Practice: Metta for oneself and for others. Informal practice: thank you letter. Task: yoga alternating with 30′ sitting + walking meditation.	
7th SESSION	Positive psychology. Values. Practice: a compassionate gesture, safe place. The funeral. Informal practice: 3 positive things, 3′. Task: 45′ method of choice.	
8th SESSION	Course feedback. Practice: review course practices. Incorporating mindfulness into daily life. Course evaluation and satisfaction questionnaire.	
POST-INTERVENTION MEASUREMENT	Repeat the questionnaires.	Repeat the questionnaires.
REEVALUATION (3 MONTHS)	Repeat the questionnaires.	Repeat the questionnaires.

The sessions are held in groups, alternating moments of silence with others of collective exploration on the best strategies to address complex and difficult situations, always looking for practical applications in the personal and professional fields. The contents of the sessions are oriented to the knowledge of mindfulness, the perception of reality, the power of emotions, the reaction to stress and emotional tension, resilience, responding to stress, using mindful communication, taking care of oneself, time management, and integrating mindfulness into everyday life.

The training programmes will be unified in the different TU and taught by the same two instructors throughout the course to avoid any variability associated with the therapist. The instructors will have proven skills through university accreditation to deliver this training and will carefully follow the interventions, using standardised and uniform methodological criteria, which will be stated in a manual.

3) CG: without intervention. Participants will only receive a 1-h information session explaining the study design and their role in the study and inviting them to complete the expected data at two time points (coinciding with the interventions in the EG8). They will pledge not to receive any intervention and will be asked not to participate in the practice of any session of full attention or meditation techniques during the study period. After the fieldwork, they will be offered the opportunity to participate in the abbreviated training programme.

### Outcomes and measures

The professionals will be asked to complete pre- and post-intervention questionnaires. The pre-intervention questionnaire will be filled out upon recruitment (baseline). The post-intervention questionnaire will be completed at 4 (EG4) or 8 weeks (EG8 and CG) after baseline and at 3 months after completion.

To measure the dependent variables or outcomes, the following instruments will be used (an additional file shows this in more detail [see Additional file 1]):

#### Primary outcome variables

- Mindfulness: The Five Facet Mindfulness Questionnaire (FFMQ), with 39 items (range: 39–195 points) and five subscales: observing, describing, acting with awareness, non-judging of inner experience, and non-reactivity to inner experience. Higher total values indicate better full mindfulness [31–33].
- Self-compassion: The Self-Compassion Scale (SCS). The abbreviated scale of the Spanish version will be used, validated by García-Campayo et al. [34, 35], with 12 items scored with a Likert scale (1 to 5), which measures how the subject usually acts towards himself in difficult times. It consists of six subscales: self-friendliness, common humanity, mindfulness, and their opposites: self-judgement, isolation, and over-identification. Higher total values indicate more self-compassion.
- Work stress: An ordinal scale ranging from 0 (no stress level) to 10 (maximum stress level) and the Perceived Stress Questionnaire (PSQ) will be used. This questionnaire, validated by Sanz-Carrillo et al. [36], evaluates six factors: Tension-Instability-Fatigue; Social Acceptance of Conflicts; Energy and Fun; Overload; Self-fulfilment Satisfaction; Fear and Anxiety. The instrument consists of 30 items that participants answer according to the frequency with which each occurs in their lives, from 1 “almost never” to 4 “almost always”. The questionnaire is evaluated for two different periods (one recently and one in the last two years or in the last year). It classifies the subject at low, medium, or high risk.
- Burnout: the Maslach Burnout Inventory-General Survey (MBI-GS) questionnaire, with 22 items (range: 0–140 points) and three subscales: emotional exhaustion (EE), with nine items and a maximum score of 54 points; depersonalisation (DP), with five items and a maximum score of 30 points; and personal fulfilment (PF), with eight items and a maximum score of 48 points [37]. This instrument, translated into Spanish, it has been validated by Gil Monte [38].
- Empathy: the Jefferson Scale of Physician Empathy (JSPE) [39, 40]. This instrument is a multidimensional scale including 3 areas: taking perspective, compassionate attention, and “ability to put oneself in the patient’s shoes”. This scale consists of 20 items, which are determined using a Likert scale of 7 points.

#### Secondary outcome variables

- Anxiety or depression disorders: the Goldberg Anxiety and Depression Scale (GADS), validated for the Spanish population [41]. This instrument has 2 subscales, each with nine questions: an anxiety subscale (questions 1–9) and a depression subscale (questions 10–18). The first 4 questions of each subscale, questions 1–4 and questions 10–13, respectively, determine whether participants should try to answer the other questions.
- Self-perceived health status will be determined by a closed question, based on the one used in the Spanish National Health Survey [42]. Likewise, the participant’s feelings of loneliness, social and work isolation, and happiness, before and after the interventions, will be assessed by two closed questions with an ordinal qualitative scale.

### Other measures/covariates

To control for potential predictor or confounding effects, the following measures will be assessed: age, sex, profession (physician or nurse), type of professional (MIR, EIR, or tutor), year of residency for residents, time as a tutor, time working in AP for tutors, and teaching unit (TU).

### Sample size

To calculate the sample size, we have drawn on a previous study [25], using as an outcome variable the mean FFMQ score [31]; accepting an alpha risk of 0.05 and a beta risk of 0.2, in a bilateral contrast, 38 subjects are required in each group to detect a difference ≥ 15 points in the FFMQ scale between both experimental groups (EG4 and EG8) and the CG. It is assumed that the common standard deviation is 20. A loss or withdrawal rate of 25% has been estimated [43]. Because this is a cluster randomisation study, we have considered the design effect. The design effect multiplies the sample size by a factor ranging from 1.5 to 3 to achieve the same power, depending on the relationship between intra- and intergroup variance [44]. Estimates of the intra-cluster correlation coefficient (ICC) in randomised clinical trials in AP are generally < 0.05 [45]. This ICC, for a cluster size of 15, results in a design effect corresponding to a factor of 1.7. Assuming this value, the final study sample size would be at least 130 subjects. One hundred and thirty-two (132) professionals will be included, 44 in each comparison group, 22 for each teaching unit.

### Recruitment

The study will be disseminated through the existing communication channels of the participating TU (mailing to the email addresses of the existing lists, blog/web, EIR websites, teaching sessions, or face-to-face meetings). After the aim of the study is explained, the professionals will be invited to take part in the study and must fill out both the commitment and informed consent forms.

### Assignment of interventions and randomisation

Cluster randomisation will be used, and each TU will be a cluster. Subject selection will be performed in each cluster and stratified by type of professional (66 tutors vs. 66 residents) between the CG (2 TU) and the two intervention groups (4 TU).

### Data collection and data management

The measurements will be taken at three time points: 1) Pre-intervention (initial or baseline; one week before the interventions begin, participants from all groups will be invited to a face-to-face meeting); 2) Post-intervention (final, one month for EG4 and two months for EG8, and simultaneously with the latter for CG participants); and 3) Three months after the interventions, at which time participants of EG4 and EG8 will be reconvened for a face-to-face session.

To measure adherence to the training programs, the sessions attended by the participants will be continuously monitored and followed up, and students must write in a personal notebook or journal whether they did the exercises at home and show it to the instructor in each session for supervision.

The data obtained (data collection notebook and questionnaires) will be collected and sent to the coordinating headquarters for further processing, debugging, and statistical analysis. Double data entry will be used for all questionnaires to keep the rate of data errors very low.

### Statistical methods

The descriptive statistics of baseline characteristics of study participants and of questionnaire results—both before and after the intervention—will include means, standard deviations and ranges, medians and interquartile ranges, or frequencies and percentages, according to the variables. The fit of quantitative variables to the normal distribution will be checked by the Kolmogorov-Smirnov test. Prior to testing the effectiveness of the intervention, appropriate statistical tests will be used to examine possible baseline differences between groups (age, sex, time worked, profession, year of residency, previous mindfulness training). A per-protocol analysis will be performed in which all subjects who have completed at least 6 of the 8 sessions in EG8, or 3 of the 4 sessions in EG4, will be considered. All signalment analyses will be intention-to-treat in order to control for non-random dropout effects. Patterns of missing values ​​will be inspected and, if applicable, replaced using appropriate statistical methods.

To compare pre- and post-intervention results, a bivariate analysis will be performed, using Student’s t-test or the Wilcoxon test to compare related samples. Pre-post differences in the results of the questionnaires between the groups will be compared using ANOVA for repeated measures. Finally, a multivariate analysis will verify the effect of the interventions, adjusting for presumably predictive and/or confounding variables. To accomplish this, a multilevel linear regression will be used. The level of statistical significance will be set at *p* < 0.05 for all statistics. The analyses will be performed with SPSS version 17.0 and MLwiN version 3.00 software.

### Quality controls, monitoring and follow-up

The educational programmes will be sent to the Spanish Health Quality Agencies for accreditation. Some sessions of the intervention groups will be randomly audio recorded, and external experts (JGC, MNG) will assess that the intervention reliably follows the protocol and includes no other interventions. To assess the lasting effect of the intervention programs, a follow-up will be conducted through a third measurement three months after the end of the programmes. At that time, participants must respond to a questionnaire to check the extent to which they have continued to perform the mindfulness practices learned. They will also fill out the FFMQ, SCS, PSC, MBI and JSPE and will be asked again about their feelings of happiness, loneliness, and social and work isolation.

### Ethical considerations

Explicit authorisation will be requested from the management of the participating TU.

This research complies with the regulatory framework of reference to develop research projects in Spain (Law 41/2002 of November 14, regulating patient autonomy, and rights and obligations regarding information and documentation). Under Law 14/2007 of Biomedical Research, on the protection of personal data and guarantee of confidentiality, the data will not be used for purposes other than those expressed in the informed consent form. The confidentiality of the information will be preserved by following the necessary regulations; data recording will respect the precepts established in the current legislation on the protection of personal data. The study will conform to the standards of good clinical practice (art. 34 RD 223/2004; European Community Directive 2001/20/EC) and the protection of personal data (Regulation (EU) 2016/679 of the European Parliament and of the Council of 27 April 2016 on the protection of natural persons with regard to the processing of personal data and on the free movement of such data-GDPR).

### Dissemination policy

In the dissemination of the study, the publication rules of the Consolidated Standards of Reporting Trials (CONSORT) Statement for the reporting of cluster randomised trials will be followed (http://www.bmj.com/content/345/bmj.e5661.long).

## Discussion

This clinical trial aims to test the effectiveness of an abbreviated intervention against a standard programme and control in reducing stress and burnout in AP health professionals with teaching functions or in the postgraduate training period. Environmental evidence allows us to show the correlation between practices to cultivate mindfulness and self-compassion and the increase in resilience and psychological well-being of AP professionals [14, 46, 47], but the effect of these educational programmes has been little studied in those health professionals who combine their care activities with teaching, which usually entails more responsibility and work stress. Montero-Marín et al. [48] have shown that not all manifestations of burnout follow the typical pattern, as some subtypes hide within a flurry of activity, simulating a situation that may seem contrary to burnout. This situation may particularly occur in professionals with additional dedication to care activities, such as those teachers who perform voluntary and altruistic tutoring in our country. This characteristic makes our trial especially relevant because it will detect situations of hidden stress in an overloaded collective.

The expected results of this trial will be immediately applicable because, if our hypothesis is confirmed, an important group of professionals—tutors and residents— who form an essential collective of the Spanish SNS will benefit. In fact, incorporating the new evidence provided by this trial into the public health service will result in a better working environment, improving the quality of care and medical practice in routine patient care. National Health Services such as the SNS—subject to a significant care overload, even more pronounced in AP—require effective, shorter, and less costly interventions to improve accessibility, acceptability, and adherence. Mindfulness programmes can be offered if we can achieve profitable and realistic formats, adjusted to the characteristics and conditions existing in different target populations [15].

### Strengths and limitations

With the cluster randomisation method, we expect to minimise the Hawthorne effect (contamination bias) in the intervention study. Withdrawals or losses may occur, which would cause a selection bias because the characteristics of the non-responders may differ from those of the responders. Due to the experience of the instructors in charge, adherence is likely to be very high at the beginning of the training program, but the loss rate thereafter could reach 20%. To lessen this problem, the sample size was increased by 25% [44]. In addition, to minimise and control this effect, from the statistical point of view, an intention-to-treat analysis will be performed in addition to the per-protocol analysis.

The questionnaires to be used are validated for the Spanish population and will be filled out anonymously (an identification code will be assigned to match those completed before and after the intervention), so we expect no problems of validity and reliability leading to important information biases.

Although the CG does not perform any type of intervention, this study does not ensure that it will remain inactive throughout the fieldwork period, which may minimise the differences in the expected results when comparing this group with the intervention groups.

To verify that the effect of the intervention programmes lasts over time, a measurement will be taken three months after their application. Although the ideal would be farther in the future, this distance is not feasible given that some participants will conclude their training period soon after completing the study, making it difficult to recruit them afterwards.

Our aim is to incorporate and extend the practice of mindfulness to the work, teaching, and care activities of health professionals—counting on the evidence to be found in this trial and contribute to increasing and complementing the countless existing tests that have been applied in other work sectors, both public and private.

## Additional file

Additional file 1: Questionnaires in Spanish and in English. The questionnaires to be used in the study, in Spanish and in English, are presented. (DOC 131 kb)

## Abbreviations

AP: Primary Care
CG: Control Group
EG4: Experimental Group, 4 weeks
EG8: Experimental Group, 8 weeks
EIR: Internal Nurse Resident
ICC: Intra-cluster correlation coefficient
MBSR: Mindfulness-Based Stress Reduction
MINDUUDD: Spanish acronym of the words Mindfulness (MIND) and Teaching Units (UUDD)
MIR: Internal Medical Resident
MSC: Mindful Self-Compassion program
SNS: National Health System
TU: Teaching Unit
