# Supplementary material for: Controlled clinical trial comparing the effectiveness of a mindfulness and self-compassion 4-session programme versus an 8-session programme to reduce work stress and burnout in family and community medicine physicians and nurses: MINDUUDD study protocol
Source: BMC Fam Pract. 2019 Feb 6;20:24. doi: 10.1186/s12875-019-0913-z (PMC6364464; doi:10.1186/s12875-019-0913-z)
Supplement: Supplementary file 1 — Questionnaires in Spanish and in English. The questionnaires to be used in the study, in Spanish and in English, are presented. (DOC 131 kb) [file 12875_2019_913_MOESM1_ESM.doc]

Annex 1. Questionnaires in Spanish and in English

Five Facets of Mindfulness Questionnaire (FFMQ-E). Spanish version

Por favor, puntué cada uno de los siguientes enunciados según la siguiente escala. Escriba el número en el espacio que mejor describa su propia opinión o lo que es verdadero de forma general para usted.

1 nunca o pocas veces/2 raras veces/3 a veces/4 a menudo/5 muy a menudo o siempre

1. Mientras camino me doy cuenta de las sensaciones de mi cuerpo al moverse.

2. Soy capaz de encontrar las palabras adecuadas para describir mis sentimientos.

3. Me critico por tener emociones irracionales o inapropiadas.

4. Percibo mis sentimientos y emociones sin tener que reaccionar ante ellas.

5. Cuando estoy haciendo algo me distraigo con facilidad.

6. Cuando me ducho o me baño, me doy cuenta de las sensaciones del agua sobre mi cuerpo.

7. Puedo expresar fácilmente con palabras mis creencias, opiniones y expectativas.

8. No presto atención a lo que hago porque estoy soñando despierto, preocupado con otras cosas o distraído.

9. Observo mis sentimientos sin enredarme en ellos.

10. Me digo a mí mismo que no debiera sentirme como me siento.

11. Me doy cuenta de cómo los alimentos y las bebidas afectan a mis pensamientos, sensaciones corporales y emociones.

12. Me resulta difícil encontrar palabras para describir lo que pienso.

13. Me distraigo fácilmente.

14. Creo que algunos de mis pensamientos no son normales o son malos y no debería pensar de esa forma.

15. Presto atención a las sensaciones, como las del viento en mi pelo o el sol sobre mi cara.

16. Tengo dificultad a la hora de pensar en las palabras adecuadas para expresar lo que siento acerca de las cosas.

17. Juzgo si mis pensamientos son buenos o malos.

18. Encuentro dificultad para estar centrado en lo que ocurre aquí y ahora, en el presente.

19. Cuando tengo pensamientos o imágenes que me perturban, “paro” y me doy cuenta sin dejarme llevar por ellos.

20. Presto atención a los sonidos, por ejemplo, al tic-tac de los relojes, al trino de los pájaros o a los coches que pasan.

21. En situaciones difíciles, puedo pararme sin reaccionar de modo inmediato.

22. Cuando tengo una sensación en mi cuerpo, me resulta difícil describirla porque no encuentro las palabras adecuadas.

23. Tengo la impresión de que “pongo el piloto automático” sin ser muy consciente de lo que estoy haciendo.

24. Cuando tengo pensamientos o imágenes que me perturban, consigo calmarme al poco tiempo.

25. Me digo a mí mismo que no debería pensar de la forma en que lo hago.

26. Me doy cuenta de los olores y aromas de las cosas.

27. Incluso cuando me siento muy contrariado, encuentro una forma de expresarlo en palabras.

28. Realizo actividades sin estar realmente atento a las mismas.

29.Cuando tengo pensamientos o imágenes que me perturban, soy capaz de darme cuenta sin reaccionar.

30. Creo que algunas de mis emociones son malas o inapropiadas y no debería sentirlas.

31. Me doy cuenta de elementos visuales en el arte o en la naturaleza tales como colores, formas, texturas, o patrones de luz y sombra.

32. Tengo una tendencia natural a expresar mis experiencias en palabras.

33. Cuando tengo imágenes o pensamientos que me perturban, reparo en ellos y dejo que se vayan.

34. Realizo trabajos o tareas de forma automática sin darme cuenta de lo que estoy haciendo.

35. Cuando tengo pensamientos o imágenes que me perturban, la valoración sobre mí mismo es buena o mala, en consonancia con el contenido de ese pensamiento o imagen.

36. Presto atención a la forma en que mis emociones afectan a mis pensamientos y comportamiento.

37. Puedo describir cómo me siento en un momento dado, de forma bastante detallada.

38. Me sorprendo a mí mismo haciendo cosas sin prestar atención.

39. Me reprocho a mí mismo cuando tengo ideas irracionales.

Reference: Cebolla A, García-Palacios A, Soler J, Guillen V, Baños R, Botella C. Psychometric properties of the Spanish validation of the Five Facets of Mindfulness Questionnaire (FFMQ) Rev Eur J Psychiat. 2012.

Five Facet Mindfulness Questionnaire (FFMQ). English version

Description: This instrument is based on a factor analytic study of five independently developed mindfulness questionnaires. The analysis yielded five factors that appear to represent elements of mindfulness as it is currently conceptualized. The five facets are observing, describing, acting with awareness, non-judging of inner experience, and non-reactivity to inner experience.

More information is available in: Please rate each of the following statements using the scale provided. Write the number in the blank that best describes your own opinion of what is generally true for you.

1 never or very rarely/2 sometimes/3 often/4 very often or rarely true/5 always

1. When I’m walking, I deliberately notice the sensations of my body moving. ___

2. I’m good at finding words to describe my feelings.

1. I criticize myself for having irrational or inappropriate emotions.
2. I perceive my feelings and emotions without having to react to them.
3. When I do things, my mind wanders off and I’m easily distracted.
4. When I take a shower or bath, I stay alert to the sensations of water on my body.
5. I can easily put my beliefs, opinions, and expectations into words.
6. I don’t pay attention to what I’m doing because I’m daydreaming, worrying, or otherwise distracted.
7. I watch my feelings without getting lost in them.
8. I tell myself I shouldn’t be feeling the way I’m feeling.
9. I notice how foods and drinks affect my thoughts, bodily sensations, and emotion
10. It’s hard for me to find the words to describe what I’m thinking.
11. I am easily distracted.
12. I believe some of my thoughts are abnormal or bad and I shouldn’t think that way.
13. I pay attention to sensations, such as the wind in my hair or sun on my face.
14. I have trouble thinking of the right words to express how I feel about things.
15. I make judgments about whether my thoughts are good or bad.
16. I find it difficult to stay focused on what’s happening in the present.
17. When I have distressing thoughts or images, I “step back” and am aware of the thought or image without getting taken over by it.
18. I pay attention to sounds, such as clocks ticking, birds chirping, or cars passing.
19. In difficult situations, I can pause without immediately reacting.
20. When I have a sensation in my body, it’s difficult for me to describe it because I can’t find the right words.
21. It seems I am “running on automatic” without much awareness of what I’m doing.
22. When I have distressing thoughts or images, I feel calm soon after.
23. I tell myself that I shouldn’t be thinking the way I’m thinking.
24. I notice the smells and aromas of things.
25. Even when I’m feeling terribly upset, I can find a way to put it into words.
26. I rush through activities without being really attentive to them.
27. When I have distressing thoughts or images. I am able just to notice them without reacting.
28. I think some of my emotions are bad or inappropriate and I shouldn’t feel them.
29. I notice visual elements in art or nature, such as colors, shapes, textures, or patterns of light and shadow.
30. My natural tendency is to put my experiences into words.
31. When I have distressing thoughts or images, I just notice them and let them go.
32. I do jobs or tasks automatically without being aware of what I’m doing.
33. When I have distressing thoughts or images, I judge myself as good or bad, depending what the thought/image is about.
34. I pay attention to how my emotions affect my thoughts and behavior.
35. I can usually describe how I feel at the moment in considerable detail.
36. I find myself doing things without paying attention.
37. I disapprove of myself when I have irrational ideas.

Reference: Baer RA, Smith GT, Hopkins J, Krietemeyer J, Toney L. Using selfreport assessment methods to explore facets of mindfulness. Assessment. 2006; 13: 27-45.

SCS-12 items. Spanish version

Cómo suelo actuar conmigo mismo/a en momentos difíciles.

Por favor, antes de responder, lea atentamente las siguientes afirmaciones. A la izquierda de cada ítem, indique con qué frecuencia actúa en la forma señalada, empleando la siguiente escala: 1 = Casi nunca a 5 Casi siempre

-SC1. Cuando fallo en algo importante para mí, me consumen los sentimientos de ineficacia.

-SC2. Trato de ser comprensivo y paciente con aquellos aspectos de mi personalidad que no me gustan.

-SC3. Cuando me sucede algo doloroso trato de mantener una visión equilibrada de la situación.

-SC4. Cuando estoy bajo/a de ánimo, tiendo a pensar que la mayoría de la gente es probablemente más feliz que yo.

-SC5. Trato de ver mis defectos como parte de la condición humana.

-SC6. Cuando lo estoy pasando verdaderamente mal, me doy el cuidado y el cariño que necesito.

-SC7. Cuando algo me disgusta, trato de mantener mis emociones en equilibrio.

-SC8. Cuando fallo en algo que es importante para mí, tiendo a sentirme solo en mi fracaso.

-SC9. Cuando me siento bajo/a de ánimo, tiendo a obsesionarme y fijarme en todo lo que va mal.

-SC10. Cuando me siento incapaz de alguna manera, trato de recordarme que casi todas las personas comparten sentimientos de incapacidad.

-SC11. Desapruebo mis propios defectos e incapacidades y soy crítico/a respecto a ellos.

-SC12. Soy intolerante e impaciente con aquellos aspectos de mi personalidad que no me gustan.

Reference: Garcia-Campayo J, Navarro-Gil M, Andrés E, Montero-Marin J, López-Artal L, Demarzo MM. Validación de las versiones en español de las formas larga (26 ítems) y corta (12 ítems) de la Escala de Auto-Compasión (SCS). Resultados de vida de vida de salud. 2014; 12: 4. Doi: 10.1186 / 1477-7525-12-4.

SCF-SF (self-compassion scale: short form). English version

How I typically act towards myself in difficult times … please read each statement carefully before answering; using the scale given below indicate, to the right of each item, how often you behave in the stated manner: almost never almost always 1 2 3 4 5

1. when I fail at something important to me I become consumed by feelings of inadequacy

2. I try to be understanding and patient towards those aspects of my personality I don't like

3. when something painful happens I try to take a balanced view of the situation

4. when I’m feeling down, I tend to feel like most other people are probably happier than I am

5. I try to see my failings as part of the human condition

6. when I’m going through a very hard time, I give myself the caring and tenderness I need

7. when something upsets me I try to keep my emotions in balance

8. when I fail at something that's important to me, I tend to feel alone in my failure

9. when I’m feeling down I tend to obsess and fixate on everything that’s wrong

10. when I feel inadequate in some way, I try to remind myself that feelings of inadequacy are shared by most people

11. I’m disapproving and judgmental about my own flaws and inadequacies

12. I’m intolerant and impatient towards those aspects of my personality

Reference: Neff KD. Development and Validation of a Scale to Measure Self-Compassion. Self and Identity. 2003; 2: 223-50

Cuestionario de estrés percibido (PSQ). Spanish versión

A.1. Instrucciones para la forma general

En cada pregunta marque un círculo en el número que mejor describa con qué frecuencia se aplica esta cuestión a su vida en general durante los últimos uno o dos años. Responda rápidamente, sin intentar comprobar las respuestas y teniendo cuidado en describir lo ocurrido en un periodo largo de tiempo.

A.2. Instrucciones para la forma reciente

En cada pregunta marque un círculo en el número que mejor describa con que´ frecuencia se aplica esta cuestión a su vida en general durante el último mes. Responda rápidamente, sin intentar comprobar las respuestas y teniendo cuidado en describir lo ocurrido exclusivamente en el último mes.

1 Casi nunca/2 A veces/3 A menudo/4 Casi siempre

(1) Se siente descansado

(2) Siente que se le hacen demasiadas peticiones

(3) Está irritable o malhumorado

(4) Tiene demasiadas cosas que hacer

(5) Se siente solo o aislado

(6) Se encuentra sometido a situaciones conflictivas

(7) Siente que esta´ haciendo cosas que realmente le gustan

(8) Se siente cansado

(9) Teme que no pueda alcanzar todas sus metas

(10) Se siente tranquilo

(11) Tiene que tomar demasiadas decisiones

(12) Se siente frustrado

(13) Se siente lleno de energía

(14) Se siente tenso

(15) Sus problemas parecen multiplicarse

(16) Siente que tiene prisa

(17) Se siente seguro y protegido

(18) Tiene muchas preocupaciones

(19) Esta bajo la presión de otras personas

(20) Se siente desanimado

(21) Se divierte

(22) Tiene miedo del futuro

(23) Siente que hace cosas por obligación, no porque quiera hacerlas

(24) Se siente criticado o juzgado

(25) Se siente alegre

(26) Se siente agotado mentalmente

(27) Tiene problemas para relajarse

(28) Se siente agobiado por la responsabilidad

(29) Tiene tiempo suficiente para usted

(30) Se siente presionado por los plazos de tiempo

Referencia: Sanz-Carrillo C, García-Campayo J, Rubio A, Santed MA, Montoro MJ. Validation of the Spanish version of the Perceived Stress Questionnaire. Psychosom Res. 2002; 52:167-72.

The Perceived Stress Questionnaire (PSQ). English version

Instructions for the General questionnaire

For each sentence, circle the number that describes how often it applies to you in general, during the last year or two. Work quickly, without bothering to check your answers, and be careful to describe your life in the long run.

Almost Sometimes Often Usually

1. You feel rested

2. You feel that too many demands are being made on you

3. You are irritable or grouchy

4. You have too many things to do

5. You feel lonely or isolated

6. You find yourself in situations of conflict

7. You feel you’re doing things you really like

8. You feel tired

9. You fear you may not manage to attain your goals

10. You feel calm

11, You have too many decisions to make

12. You feel frustrated

13. You are full of energy

14. You feel tense

15. Your problems seem to be piling up

16. You feel you’re in a hurry

17. You feel safe and protected

18. You have many worries

19. You are under pressure from other people

20. You feel discouraged

21. You enjoy yourself

22. You are afraid for the future

23. You feel you’re doing things because you have to not because you want to

24. You feel criticized or judged

25. You are lighthearted

26. You feel mentally exhausted

27. You have trouble relaxing

28. You feel loaded down with responsibility

29. You have enough time for yourself

30. You feel under pressure from deadlines

Reference: Levenstein S, Prantera V, Varvo V, Scribano ML, Berto E, Luzi C, Andreoli A. Development of the perceived Stress Questionnaire: a new tool for psychosomatic research. J Psychosom Res 1993;37:19-32.

Maslach Burnout Inventory (MBI). English version

1 I feel emotionally drained by my work

2 I feel used up at the end of the day

3 I feel fatigued when I have to get up in the morning to face another day on the job

4 I can easily understand how my recipients feel about things

5 I feel I treat some recipients as impersonal ‘objects’

6 Working with people all day is really a strain for me

7 I deal very effectively with the problems of my recipients

8 I feel ‘burned out’ from my work

9 I feel I’m a positive influence on other people’s lives through my work

10 I have become more callous toward people since I took this job

11 I worry that this job is hardening me emotionally

12 I feel frustrated by my job

13 I feel I’m working too hard in my job

14 I don’t really care what happens to some recipients

15 I can easily create a relaxed atmosphere with my recipients

16 I feel exhilarated after working with my recipients

17 I have accomplished many worthwhile things in this job

18 I feel like I’m at the end of my rope

19 In my work I deal with emotional problems calmly

20 I feel some recipients blame me for some of their problems

21 In my work, people bother me with personal problems that I don’t want to be bothered with

22 I try to keep away from the personal problems of my recipients

Reference: Maslach C, Jackson SE, Leiter MP. Maslach Burnout Inventory Manual. 3rd ed. Consulting Psychologists Press. Palo Alto, CA, 1996.

MBI (Inventario de Burnout de Maslach). Spanish versión.

| A continuación encontrará una serie de enunciados acerca de su trabajo y de sus sentimientos en él. Le pedimos su colaboración respondiendo a ellos como lo siente. No existen respuestas mejores o peores, la respuesta correcta es aquella que expresa verídicamente su propia existencia. Los resultados de este cuestionario son estrictamente confidenciales y en ningún caso accesibles a otras personas. Su objeto es contribuir al conocimiento de las condiciones de su trabajo y mejorar su nivel de satisfacción.  A cada una de las frases debe responder expresando la frecuencia con que tiene ese sentimiento de la siguiente forma: | |
| --- | --- |
| Nunca:....................................... 1 | Algunas veces al año:........................ 2 |
| Algunas veces al mes:.............. 3 | Algunas veces a la semana:.............. 4 |
| Diariamente:.............................. 5 |  |
| Por favor, señale el número que considere más adecuado: | |
| 1. AE Me siento emocionalmente defraudado en mi trabajo. | |
| 2. AE Cuando termino mi jornada de trabajo me siento agotado. | |
| 3. AE Cuando me levanto por la mañana y me enfrento a otra jornada de trabajo me siento agotado. | |
| 4. RP Siento que puedo entender fácilmente a las personas que tengo que atender. | |
| 5. D Siento que estoy tratando a algunos beneficiados de mí como si fuesen objetos impersonales. | |
| 6. AE Siento que trabajar todo el día con la gente me cansa. | |
| 7. RP Siento que trato con mucha efectividad los problemas de las personas a las que tengo que atender. | |
| 8. AE Siento que mi trabajo me está desgastando. | |
| 9. RP Siento que estoy influyendo positivamente en las vidas de otras personas a través de mi trabajo. | |
| 10. D Siento que me he hecho más duro con la gente. | |
| 11. D Me preocupa que este trabajo me esté endureciendo emocionalmente. | |
| 12. RP Me siento muy enérgico en mi trabajo. | |
| 13. AE Me siento frustrado por el trabajo. | |
| 14. AE Siento que estoy demasiado tiempo en mi trabajo. | |
| 15. D Siento que realmente no me importa lo que les ocurra a las personas a las que tengo que atender profesionalmente. | |
| 16. AE Siento que trabajar en contacto directo con la gente me cansa. | |
| 17. RP Siento que puedo crear con facilidad un clima agradable en mi trabajo. | |
| 18. RP Me siento estimulado después de haber trabajado íntimamente con quienes tengo que atender. | |
| 19. RP Creo que consigo muchas cosas valiosas en este trabajo. | |
| 20. AE Me siento como si estuviera al límite de mis posibilidades. | |
| 21. RP Siento que en mi trabajo los problemas emocionales son tratados de forma adecuada. | |
| 22. D Me parece que los beneficiarios de mi trabajo me culpan de algunos de sus problemas. | |
| AE: Agotamiento Emocional; D: Despersonalización; RP: Realización Personal. | |
| Reference: Gil Monte P. Validez factorial de la adaptación al español del Maslach Burnout Survey. Salud Publica Mex. 2002; 44: 33-40. | |

Jefferson Scale of Physician Empathy health professionals version (JSE-HP), adapted to Spanish

| 1. Comprender cómo se sienten mis pacientes y sus familiares no es un factor relevante para el tratamiento médico. | 1 2 3 4 5 6 7 |
| --- | --- |
| 2. Mis pacientes se sienten mejor cuando comprendo sus sentimientos. | 1 2 3 4 5 6 7 |
| 3. Me resulta difícil ver las cosas desde la perspectiva de mis pacientes. | 1 2 3 4 5 6 7 |
| 4. Considero que entender el lenguaje corporal de mis pacientes es tan importante como la comunicación verbal en las relaciones médico-paciente. | 1 2 3 4 5 6 7 |
| 5. Tengo un buen sentido del humor, lo que creo que contribuye a obtener un mejor resultado clínico. | 1 2 3 4 5 6 7 |
| 6. Como todo el mundo es diferente, me resulta casi imposible ver las cosas desde la perspectiva de los pacientes. | 1 2 3 4 5 6 7 |
| 7. Trato de no prestar atención a las emociones de mis pacientes cuando los entrevisto y hago su historia clínica. | 1 2 3 4 5 6 7 |
| 8. Prestar atención a las experiencias personales de mis pacientes es irrelevante para la efectividad del tratamiento. | 1 2 3 4 5 6 7 |
| 9. Trato de ponerme en el lugar de mis pacientes cuando les atiendo. | 1 2 3 4 5 6 7 |
| 10. El hecho de comprender los sentimientos de mis pacientes les aporta una sensación de reconocimiento que resulta terapéutica por sí misma | 1 2 3 4 5 6 7 |
| 11. Las enfermedades de los pacientes sólo se pueden curar mediante tratamiento médico y, por tanto, ningún vínculo afectivo que establezca con mis pacientes podrá tener un valor significativo en ese cometido. | 1 2 3 4 5 6 7 |
| 12. Considero que preguntar a mis pacientes sobre lo que sucede en sus vidas es un factor sin importancia para comprender sus quejas físicas. | 1 2 3 4 5 6 7 |
| 13. Trato de comprender qué pasa por la mente de mis pacientes prestando atención a su comunicación no verbal y a su lenguaje corporal. | 1 2 3 4 5 6 7 |
| 14. Creo que no hay lugar para las emociones en el tratamiento de las enfermedades médicas. | 1 2 3 4 5 6 7 |
| 15. La empatía es una destreza terapéutica sin la cual mi éxito como médico estaría limitado. | 1 2 3 4 5 6 7 |
| 16. Un componente importante de la relación con mis pacientes es mi comprensión de su estado emocional y el de sus familiares. | 1 2 3 4 5 6 7 |
| 17. Trato de pensar como mis pacientes para prestarles una mejor atención. | 1 2 3 4 5 6 7 |
| 18. No permito que me afecten las relaciones emocionales intensas entre mis pacientes y sus familiares. | 1 2 3 4 5 6 7 |
| 19. No disfruto leyendo literatura no médica ni con actividades artísticas. | 1 2 3 4 5 6 7 |
| 20. Creo que la empatía es un factor terapéutico importante en el tratamiento médico. | 1 2 3 4 5 6 7 |

JSE-HP, English version, adapted to Spanish

| 1. My understanding of how my patients and their relatives feel is an irrelevant factor to medical treatment. | 1 2 3 4 5 6 7 |
| --- | --- |
| 2. My patients feel better when I understand their feelings. | 1 2 3 4 5 6 7 |
| 3. I find it difficult to see things from my patients’ perspective. | 1 2 3 4 5 6 7 |
| 4. I believe that understanding my patients’ body language is as important as verbal communication in doctor-patient relationships. | 1 2 3 4 5 6 7 |
| 5. I have a good sense of humour, an aspect I think contributes to obtaining a better clinical outcome. | 1 2 3 4 5 6 7 |
| 6. Since everyone is different, I find it almost impossible to see things from my patients’ perspective. | 1 2 3 4 5 6 7 |
| 7. I try not to pay attention to my patients’ emotions when I interview them and write down their medical record. | 1 2 3 4 5 6 7 |
| 8. Paying attention to my patients’ personal experiences is irrelevant to treatment effectiveness. | 1 2 3 4 5 6 7 |
| 9. I try to put myself in my patients’ shoes when seeing them. | 1 2 3 4 5 6 7 |
| 10. Understanding my patients’ feelings gives them a sense of validation that is therapeutic in itself. | 1 2 3 4 5 6 7 |
| 11. Patients’ illnesses can only be cured by medical treatment and, therefore, no emotional bond established with my patients will have any significant value in that task. | 1 2 3 4 5 6 7 |
| 12. I believe that asking my patients about what is going on in their lives is an unimportant factor in understanding their physical complaints. | 1 2 3 4 5 6 7 |
| 13. I try to understand what is going on in my patients’ mind by paying attention their non-verbal communication and body language. | 1 2 3 4 5 6 7 |
| 14. I don’t think there is any room for emotions in the treatment of medical illnesses. | 1 2 3 4 5 6 7 |
| 15. Empathy is a therapeutic skill without which my success as a doctor would be limited. | 1 2 3 4 5 6 7 |
| 16. An important component of the relationship with my patients is my understanding of their emotional state and that of their relatives. | 1 2 3 4 5 6 7 |
| 17. I try to think like my patients to give them better care. | 1 2 3 4 5 6 7 |
| 18. I don’t let intense emotional relations between my patients and their relatives affect me. | 1 2 3 4 5 6 7 |
| 19. I don’t enjoy reading literature that is not medical or with experiences that are not related to art. | 1 2 3 4 5 6 7 |
| 20. I believe empathy is an important therapeutic factor in medical treatment. | 1 2 3 4 5 6 7 |

© Thomas Jefferson University. All rights reserved.

Reference: Blanco JM, Caballero F, García FJ, Lorenzo F, Monge D. Validation of the Jefferson Scale of Physician Empathy in Spanish medical students who participated in an Early Clerkship Immersion programme. BMC Medical Education. 2018; 18: 209. https://doi.org/10.1186/s12909-018-1309-9.

Escala de Ansiedad y Depresión de Goldberg (EADG). Spanish versión

Subescala de ansiedad

1. ¿Se ha sentido muy excitado, nervioso o en tensión?

2. ¿Ha estado muy preocupado por algo?

3. ¿Se ha sentido muy irritable?

4. ¿Ha tenido dificultad para relajarse?

(Si hay 3 o más respuestas afirmativas, continuar

preguntando)

5. ¿Ha dormido mal, ha tenido dificultades para dormir?

6. ¿Ha tenido dolores de cabeza o de nuca?

7. ¿Ha tenido alguno de los siguientes síntomas: temblores,

hormigueos, mareos, sudores, diarrea? (síntomas

vegetativos)

8. ¿Ha estado preocupado por su salud?

9. ¿Ha tenido alguna dificultad para conciliar el sueño, para

quedarse dormido?

Total ansiedad= _______

Subescala de depresión

1. ¿Se ha sentido con poca energía?

2. ¿Ha perdido Vd. el interés por las cosas?

3. ¿Ha perdido la confianza en sí mismo?

4. ¿Se ha sentido Vd. desesperanzado, sin esperanzas?

(Si hay respuestas afirmativas a cualquiera de las preguntas

anteriores, continuar)

5. ¿Ha tenido dificultades para concentrarse?

6. ¿Ha perdido peso? (a causa de su falta de apetito)

7. ¿Se ha estado despertando demasiado temprano?

8. ¿Se ha sentido Vd. enlentecido?

9. ¿Cree Vd. que ha tenido tendencia a encontrarse peor por

las mañanas?

Total depresión=_______

Reference: Montón C, Pérez-Echevarría MJ, Campos R. Escalas de ansiedad y depresión de Goldberg: una guía de entrevista eficaz para la detección del malestar psíquico. [Goldberg anxiety and depression scales: an effective interview guide for the detection of psychological distress] Aten Primaria. 1993;12: 345-49.

Goldberg Anxiety and Depression Scale (GADS). English version

Anxiety scale

(Score one point for each "Yes")

1 Have you felt keyed up, on edge?

2 Have you been worrying a lot?

3 Have you been irritable?

4 Have you had difficulty relaxing?

(If "Yes" to two of the above, go on to ask:)

5 Have you been sleeping poorly?

6 Have you had headaches or neck aches?

7 Have you had any of the following:

trembling, tingling, dizzy spells, sweating,

frequency, diarrhoea?

8 Have you been worried about your health?

9 Have you had difficulty falling asleep?

Depression scale

(Score one point for each "Yes")

1 Have you had low energy?

2 Have you had loss of interests?

3 Have you lost confidence in yourself?

4 Have you felt hopeless?

(If "Yes" to ANY question, go on to ask:)

5 Have you had difficulty concentrating?

6 Have you lost weight (due to poor appetite)?

7 Have you been waking early?

8 Have you felt slowed up?

9 Have you tended to feel worse in the mornings?

Reference: Goldberg D, Bridges K, Duncan-Jones P, et al. Detecting anxiety and depression in general medical settings, BMJ. 1988; 297: 897-9)
